# A desirable transgenic strategy using GGTA1 endogenous promoter-mediated knock-in for xenotransplantation model

**DOI:** 10.1038/s41598-022-13536-z

**Published:** 2022-06-10

**Authors:** Nayoung Ko, Joohyun Shim, Hyoung-Joo Kim, Yongjin Lee, Jae-Kyung Park, Kyungmin Kwak, Jeong-Woong Lee, Dong-Il Jin, Hyunil Kim, Kimyung Choi

**Affiliations:** 1 Department of Transgenic Animal Research, Optipharm, Inc., Chungcheongbuk-do, Cheongju-si, 28158 Republic of Korea; 2 Department of Animal Science and Biotechnology, Chungnam National University, Daejeon, Republic of Korea; 3 Biotherapeutics Translational Research Center, Korea Research Institute of Bioscience and Biotechnology, Dajeon, Republic of Korea

**Keywords:** Biological techniques, Biotechnology, Genetics, Immunology, Molecular biology

## Abstract

Pig-to-human organ transplantation is a feasible solution to resolve the shortage of organ donors for patients that wait for transplantation. To overcome immunological rejection, which is the main hurdle in pig-to-human xenotransplantation, various engineered transgenic pigs have been developed. Ablation of xeno-reactive antigens, especially the 1,3-Gal epitope (GalT), which causes hyperacute rejection, and insertion of complement regulatory protein genes, such as hCD46, hCD55, and hCD59, and genes to regulate the coagulation pathway or immune cell-mediated rejection may be required for an ideal xenotransplantation model. However, the technique for stable and efficient expression of multi-transgenes has not yet been settled to develop a suitable xenotransplantation model. To develop a stable and efficient transgenic system, we knocked-in internal ribosome entry sites (IRES)-mediated transgenes into the α 1,3-galactosyltransferase (GGTA1) locus so that expression of these transgenes would be controlled by the GGTA1 endogenous promoter. We constructed an IRES-based polycistronic hCD55/hCD39 knock-in vector to target exon4 of the GGTA1 gene. The hCD55/hCD39 knock-in vector and CRISPR/Cas9 to target exon4 of the GGTA1 gene were co-transfected into white yucatan miniature pig fibroblasts. After transfection, hCD39 expressed cells were sorted by FACS. Targeted colonies were verified using targeting PCR and FACS analysis, and used as donors for somatic cell nuclear transfer. Expression of GalT, hCD55, and hCD39 was analyzed by FACS and western blotting. Human complement-mediated cytotoxicity and human antibody binding assays were conducted on peripheral blood mononuclear cells (PBMCs) and red blood cells (RBCs), and deposition of C3 by incubation with human complement serum and platelet aggregation were analyzed in GGTA1 knock-out (GTKO)/CD55/CD39 pig cells. We obtained six targeted colonies with high efficiency of targeting (42.8% of efficiency). Selected colony and transgenic pigs showed abundant expression of targeted genes (hCD55 and hCD39). Knocked-in transgenes were expressed in various cell types under the control of the GGTA1 endogenous promoter in GTKO/CD55/CD39 pig and IRES was sufficient to express downstream expression of the transgene. Human IgG and IgM binding decreased in GTKO/CD55/CD39 pig and GTKO compared to wild-type pig PBMCs and RBCs. The human complement-mediated cytotoxicity of RBCs and PBMCs decreased in GTKO/CD55/CD39 pig compared to cells from GTKO pig. C3 was also deposited less in GTKO/CD55/CD39 pig cells than wild-type pig cells. The platelet aggregation was delayed by hCD39 expression in GTKO/CD55/CD39 pig. In the current study, knock-in into the GGTA1 locus and GGTA1 endogenous promoter-mediated expression of transgenes are an appropriable strategy for effective and stable expression of multi-transgenes. The IRES-based polycistronic transgene vector system also caused sufficient expression of both hCD55 and hCD39. Furthermore, co-transfection of CRISPR/Cas9 and the knock-in vector not only increased the knock-in efficiency but also induced null for GalT by CRISPR/Cas9-mediated double-stranded break of the target site. As shown in human complement-mediated lysis and human antibody binding to GTKO/CD55/CD39 transgenic pig cells, expression of hCD55 and hCD39 with ablation of GalT prevents an effective immunological reaction in vitro. As a consequence, our technique to produce multi-transgenic pigs could improve the development of a suitable xenotransplantation model, and the GTKO/CD55/CD39 pig developed could prolong the survival of pig-to-primate xenotransplant recipients.

## Introduction

Xenotransplantation is a powerful source to solve the shortage of organ transplant donors. In pig-to-human xenotransplantation, ablation of the alpha 1,3-Gal epitope (GalT) synthesized by α 1,3-galactosyltransferase (GGTA1) gene is essential to inhibit hyperacute rejection, which is the most severe barrier to xenotransplantation. Since the GGTA1 knock-out (GTKO) pig was developed^1^, the survival days of heart and kidney pig-to-primate xenotransplantation were prolonged from 2 to 6 months^2–4^. Although hyperacute rejection was inhibited in GTKO pigs, early graft failure was observed 3 days after pig-to-non-human primate organ transplantation^5^. To overcome early graft failure after GTKO pig-to-non-human primate organ transplantation, additional knock-out of cytidine monophosphate-N-acetylneuraminic acid hydroxylase (CMAH) synthesizing N-glycolylneuraminic acid (Neu5Gc) and beta‐1,4‐N‐acetyl‐galactosaminyltransferase 2 (β4GalNT2) synthesizing Sda antigen have been suggested^6–10^. In spite of the double or triple knock-out of these genes in pigs, delayed graft rejection characterized by complement activation and thrombotic microangiopathy has still persisted after pig-to-non-human cardiac or renal xenotransplantation^11–13^. These findings suggest that modulation of complement activation and coagulation may be cooperatively needed with knock-out of xeno-antigens.

Transgenic pigs for complement regulatory protein such as hCD46, hCD55, and hCD59 were developed to overcome complement-mediated cytotoxicity or early graft failure compared to wild-type pigs or GTKO pigs^5,14–17^. GTKO/hCD46/hTBM pigs showed the longest survival (945 days for heterotopic, 195 days for orthotopic) in cardiac pig-to-primate xenografts^18,19^.

CD55 (decay-accelerating factor, DAF) can inhibit the complement cascade by dissociating C3 convertase into its constituent proteins^20,21^. Several convincing studies have shown that hCD55 is an applicable complement regulatory protein to protect against complement-mediated injury in xenografts^22–24^. The transgenic porcine heart with only hDAF was life-supported for 39 days, and GTKO/hCD55 transgenic pigs survived over 400 days in pig-to-non-human primate renal xenotransplantation^17,25^. Furthermore, recent research found that deficiency of hCD55 is related to the occurrence of angiopathic thrombosis^26^.

CD39 (ectonucleoside triphosphate diphosphohydrolase-1, ENTPD1) is one of the thromboregulatory molecules expressed on the luminal surface and caveolar microdomains of quiescent endothelial cells^27,28^, and is able to prevent coagulation by inactivating platelet aggregation. Platelet aggregation is stimulated when the endothelium is damaged from immunological rejection. CD39 has the role of NTPDase to regulate platelet aggregation. CD39 converts ADP, which is a potent agonist to trigger platelet aggregation, to AMP, and AMP is broken down by ecto-5’-nucleotidase (CD73) to adenosine^27,29^. Generated adenosine acts by inactivating platelet aggregation.

For these multiple immune-protective effect in pig-to-human xenotransplantation, many attempts using the internal ribosome entry sites (IRES) and the self-cleavable 2A sequence, which are widely studied for basic science, and therapeutic research, have been developed for the production of multi-transgenic pigs^30–33^. Recently, knock-out of four genes/transgenic for nine genes was developed using 2A and the piggy-bag mediated integration system^34^.

Over expression of transgenes is a fundamental option across the broad scientific research using genetically engineering animal models. Induction of transgene expression can be applied to study gene function, gene therapy, and biopharmaceuticals^35–37^. Despite these various uses for the expression of transgenes, transfection by a plasmid, lentiviral, and transposon systems have still shown to induce gene silencing, or insertional mutagenesis, or unknown chromosome position effects, or position-effect variegations^38–42^. Exogenous promoters for the expression of transgenes also results in gene silencing of the transgene^43–49^. Multi-transgenic pigs could be produced by breeding of each single gene expressed transgenic pigs, but it is not efficient in costs and time, and it is unpredictable. In addition, multi-transgenic pigs engineered using IRES-mediated tricistronic vector systems showed pretty low expression of the third gene of the inserted vector^32^.

On the contrary, targeting of the transgene into specific genome locus revealed abundant expression of transgene^50–52^, and expression of the transgene under the control of endogenous promoters of target gene loci for knock-in revealed stable expression over a long time^53,54^.

The objective of this report is to demonstrate that endogenous GGTA1 promoter-mediated multi-transgene expression by gene-targeting was stable and effective to create a transgenic pig model aimed at preventing immunological rejection towards humans in vitro.

## Results

### Construction of GTKO/CD55/CD39 knock-in vector and Establishment of targeted donor cell.

A total 785 reconstructed embryos derived from the GTKO/CD55/CD39 donor cells were transferred into four surrogates. Two surrogates became pregnant, and one of those surrogates gave birth to four live born piglets, and one piglet was survived (supplementary Fig. S4). The cloning efficiency was 2.1% in this single transfer. In total, the cloning efficiency was 0.5% (total piglets/total transferred embryos) (Table 1). The targeting strategy to knock-out the GGTA1 gene and knock-in the hCD55 and hCD39 genes and guide RNA sequences, to target initiation codon in exon 4 of the GGTA1 gene, were designed as described in supplementary Fig. S1A and S1B. Two homologous arms were designed to target exon 4, which contains the ATG initiation codon. Between two homologous arms, the cDNA for hCD55 and hCD39 were inserted under control of the GGTA1 endogenous promoter. We chose electroporation, which shows high efficiency of transfection for primary cultured cells, such as ear skin fibroblasts to transfect targeting vectors into pig ear skin fibroblasts. FACS sorting using hCD39 expression was performed to select targeted cells because we expected that only targeted cells would express hCD39. hCD39 positive cells were 20% of the total cells and sorted (supplementary Fig. 1C). After selection using FACS sorting, 24 single colonies were picked and 14 colonies were analyzed to verify targeting through targeting PCR (supplementary Fig. 1D). In six colonies the vector was successfully targeted on exon 4 of the GGTA1 gene with high efficiency (42.8% of efficiency) (Table 2). Colony #15 was used as donor for somatic nuclear transfer (SCNT).

**Table 1:** In vivo developmental potential of somatic cell nuclear transfer (SCNT) embryos reconstructed using GTKO/CD55/CD39 transgenic donor cells.

Donor cells	No. of SCNT embryos transferred (embryos per transfer)	No. of recipients	Pregnancy (%)^a^	Delivery (%)	Produced piglets (survived)	Average birth weight of piglets (g)	Cloning efficiency in single transfer/total (%)^b^
#15	785 (196)	4	2 (50)	1 (50)	4 (1)	566.5	2.1/0.5

^a^Pregnant recipients/total recipients.

^b^piglets/total embryos transferred.

**Table 2:** Efficiency of GTKO/CD55/CD39 knock-in vector targeted into GGTA1 locus.

No. of picked colonies	No. of analyzed colonies	No. of right arm (+) colonies (%)	No. of left arm (+) colonies (%)	No. of long PCR (+) colonies (%)	No. of targeted colonies (%)
24	14	13 (92.8)	13 (92.8)	6 (42.8)	6 (42.8)

### Ablation of GalT and GGTA1 endogenous promoter-mediated expression of hCD55 and hCD39

**Figure 1:**
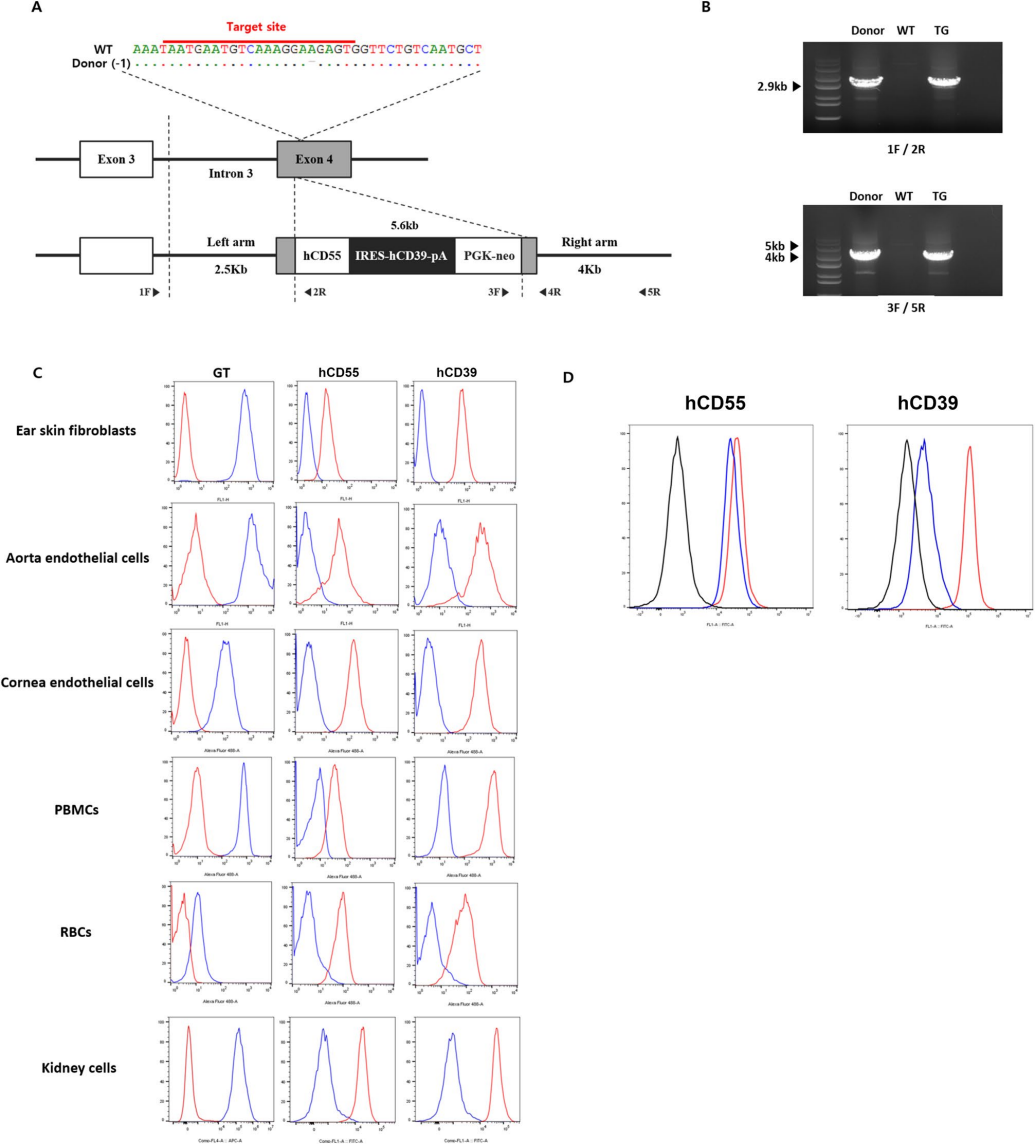
Ablation of GalT and targeted hCD55 and hCD39 gene at GGTA1 locus on produced GTKO/CD55/CD39 targeted cloned pig. (**A**) Schematic of targeting strategy to ablate GalT and knock-in of hCD55 and hCD39 gene. (**B**) Targeting PCR analysis in GTKO/CD55/CD39 transgenic pig (Donor; targeted colony, WT; wild-type, TG; transgenic pig). (**C**) Cell surface expression of GalT, hCD55, and hCD39 on variety cell source from wild type (blue line) and GTKO/CD55/CD39 transgenic pig (red line). (**D**) Comparison of hCD55 and hCD39 expression on GTKO/CD55/CD39 pig aorta endothelial cells (red line), and human aorta endothelial cells (blue line). Wild-type pig aorta endothelial cells (black line) was used as negative control. The original gels are presented in supplementary Fig. S3.

We selected one of the targeted six colonies, which had greater cell morphology and proliferation. hCD55 and hCD39 were abundantly expressed on the cell surface of targeted donor cells and expression of the proteins was also confirmed (supplementary Fig. S1E and S1F). The targeting vector was knocked-in to the one allele of the GGTA1 locus, with consequent heterozygous knock-out of the GGTA1 gene. However, the other allele which was not knocked-in was also disrupted by CRISPR/Cas9, thereby inducing homozygous knock-out of the GGTA1 gene (Fig. 1A and supplementary Fig. S4C).

### Production of GTKO/CD55/CD39 knock-in targeted transgenic cloned pig

Cloned piglets possessing the targeting vector inserted into the GGTA1 locus were produced and their genotype verified by targeting PCR (Fig. 1B). hCD55 and hCD39 was abundantly expressed in ear skin fibroblasts cells, aorta endothelial cells, cornea endothelial cells, peripheral blood mononuclear cells (PBMCs), red blood cells (RBCs), and kidney cells from GTKO/CD55/CD39 transgenic pig. The transgenic animals also revealed ablation of the aGal epitope on the surface of these cells (Fig. 1C). Moreover, the expression level of hCD55 was similar between human and GTKO/CD55/CD39 pig aorta endothelial cells. In the case of hCD39, the expression level in GTKO/CD55/CD39 pig aorta endothelial cells was higher than in human endothelial cells (Fig. 1D).

### Human antibody binding

**Figure 2:**
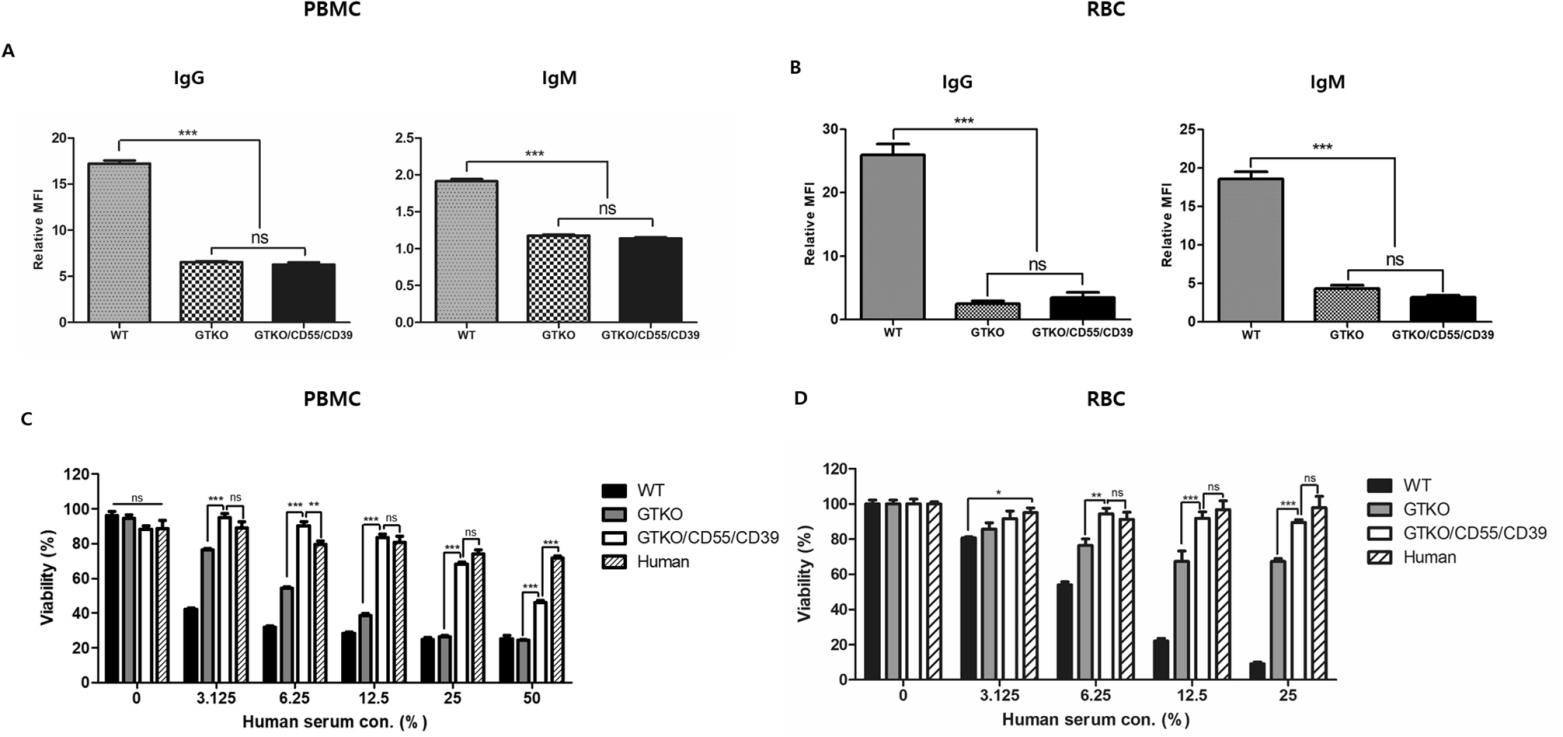
Prevention of humoral rejection on GTKO/CD55/CD39 pig in vitro. Human antibody binding to PBMCs (**A**) and RBCs (**B**). Both human IgG and IgM binding to cells from GTKO and GTKO/CD55/CD39 pig decreased compared with cells from wild-type pig. Data represented relative mean fluorescence intesnsity (MFI). Experiments were performed in triplicated (n = 1, ***P < 0.05, one-way analysis of variance). Inhibition of human complement mediated rejection on cells from GTKO/CD55/CD39 pig. PBMCs (**C**) and RBCs (**D**) from wild-type, GTKO/CD55/CD39 pig and human. These cells were incubated with various human complement serum concentration, and the viability was represented by calculation with absorbance of CCK-8 (PBMCs) or viable remaining cells (RBCs). Experiments were performed in triplicated (n = 1; * P < 0.05; **P < 0.01; ***P < 0.001; ns, non-significant; two-way analysis of variance).

Human antibody binding to PBMCs and RBCs from GTKO/CD55/CD39 pigs was greatly reduced compared with binding to PBMCs and RBCs from wild-type pigs. However, human antibody binding to PBMCs (Fig. 2A) and RBCs (Fig. 2B) was not significantly different between GTKO and GTKO/CD55/CD39 pig.

### Inhibition of immunological rejection by human complement

The viability against human complement was estimated using peripheral blood mononuclear cells (PBMCs) and red blood cells (RBCs) from wild-type, GTKO, and GTKO/CD55/CD39 pig. Both GTKO/CD55/CD39 cell types remarkably prevented human complement-mediated cytotoxicity against various human complement serum concentration (0%, 3.125%, 6.25%, 12.5%, and 25%) compared with wild-type and GTKO cells. Against most concentrations of human complement serum, the viability of PBMCs and RBCs from GTKO/CD55/CD39 pig was not significantly different from that of human PBMCs or RBCs. As shown in Fig. 2C, PBMCs from wild-type and GTKO pigs survived only 24.95–42.09% (wild-type), and 24.19–76.28% (GTKO), whereas the viability was 45.81–94.72% against various human complement serum concentration in PBMCs from GTKO/CD55/CD39 (Fig. 2C). As shown in Fig. 2D, the mean viability of wild-type pig RBCs against human complement serum was only 9.1–80.6%. The mean viability of GTKO pig RBCs was 67.25–85.7%, and that of GTKO/CD55/CD39 pig RBCs was significantly higher (89.3–91.5%).

### C3 deposition

**Figure 3:**
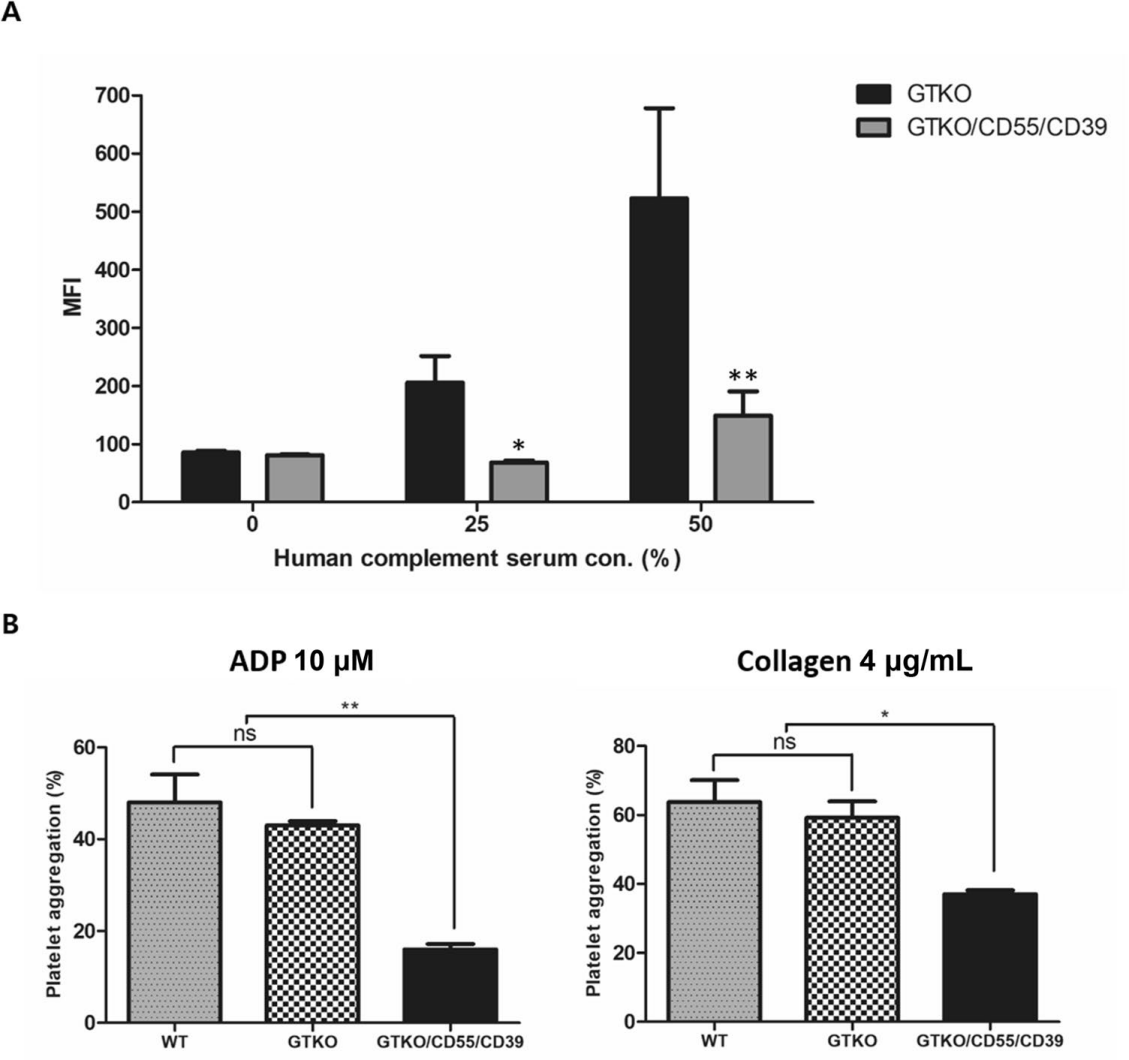
Functional hCD55 and hCD39 gene expressed on GTKO/CD55/CD39 pig. (**A**) Decrease of C3 deposition on RBCs from GTKO/CD55/CD39 compared with RBCs from GTKO pig at 0, 25, and 50% of human complement serum concentration. Experiments were performed in triplicated (n = 1, t-test; *P < 0.05; **P < 0.01; ns, non-significant). (**B**) Inhibition of platelet aggregation in GTKO/CD55/CD39 pig by platelet aggregation test response to ADP (10 µM) and collagen (2 mg/mL). Experiments were performed in independently triplicated (n = 1, *P value = 0.0169; **P value = 0.0014, one-way analysis of variance).

Deposition of C3 was estimated following activation of complement through incubation of RBCs with 0%, 25%, and 50% of human complement serum. MFI of C3 on RBCs from GTKO/CD55/CD39 pigs were 81.26–149 at 0, 25, and 50% concentration, whereas mean fluorescence intensity (MFI) of C3 on RBCs from GTKO were 85.7–523 at 0, 25, and 50% concentration, respectively. It was impossible to detect C3 deposition in RBCs from wild-type due to the dramatic hemolysis which occurred following human complement activation (Fig. 3A).

### Platelet aggregation

Platelet aggregation was tested in wild-type and GTKO/CD55/CD39 transgenic pigs’ PRP.

The maximal aggregation of platelets in wild-type and GTKO pigs was between 38 and 64%, whereas maximal aggregation of platelets was only 14–18% in GTKO/CD55/CD39 pigs against 10 µM of ADP. Against collagen (4 µg/mL), the maximal aggregation of platelets in GTKO/CD55/CD39 pigs was only 35–39% (47 and 78% aggregation were estimated in wild-type and GTKO pigs) (Fig. 3B).

## Discussion

Production of transgenic pigs is an imperative technology for the success of pig-to-human xenotransplantation. Many genes have to be engineered to overcome various immunological rejection issues in pig-to-human xenotransplantation. Many transgenic pigs with single gene or multi-transgenic pigs with more than three engineered genes were developed in the xenotransplantation field^30,33,55,56^. Recently, the successful first clinical heart and kidney xenotransplantation^57^ was carried out using transgenic pig with ten genetic modification. Nevertheless, an effective transgenic system has not been agreed upon. In our previous study^52^, we chose the GGTA1 locus and a gene trap strategy for transgene expression due to stable and various expression of GalT^58–62^. As a result, our transgenic pig expressed the transgene abundantly without an exogenous promoter, and the transgene was well maintained in expression for several generations. Therefore, we tried to produce multi-transgenic pigs by a gene trap strategy targeting the GGTA1 locus, for the stable and efficient expression of transgenes to solve the issues with exogenous promoter-mediated transgene expression and random integration of transgenes.

The homologous recombination strategy is an effective method for gene modification in mouse embryonic stem cells by knock-out and knock-in^63,64^. Since gene-editing technologies such as ZFN, TALEN, and CRISPR/Cas9 have been developed, some studies have attempted to use TALEN or CRISPR/Cas9 systems to increase the efficiency of homologous recombination-mediated knock-in^65–68^. In this sense, we developed a system that combined CRISPR/Cas9 and a hCD55/hCD39 knock-in vector to increase efficiency. We chose to target exon 4, which includes the initiation codon of the GGTA1 gene, so that expression of hCD55 and hCD39 would be under the control of the GGTA1 gene endogenous promoter, due to the wide expression of GalT in pig, and to induce the stable expression of the transgene. We constructed a targeting vector to ablate the GGTA1 gene and express hCD55 and hCD39 by the gene trap principle. The combination of CRISPR/Cas9 and knock-in systems increased the frequency of homologous recombination, so that the targeted gene was detectable by FACS sorting (20% expression of hCD39 after neomycin selection, supplementary Fig. 1C). After FACS sorting, we obtained six precise targeted colonies of a total of 20 colonies analyzed (42.8% efficiency), with a much higher efficiency than in our previous study (2.6% of efficiency)^52^ (Table 2). Furthermore, another allele apart from the targeted allele was also mutated by the CRISPR/Cas9 effect, whereas knock-in usually resulted in heterozygous targeting (Fig. 1A and supplementary Fig. S4C).

To overcome disadvantage such as gene silencing, insertional mutagenesis, unknown chromosome position effects and position-effect variegation of random integration or exogenous promoter-mediated expression of transgene^38–49^, we chose the endogenous promoter-mediated method. The accurate targeting of transgene is essential for endogenous promoter-mediated expression. The results of our study, precisely targeting of hCD55/hCD39 knock-in vector is confirmed. However, additional review on the number of copies is required.

Anti-GalT antibodies are naturally developed in human in the first few months of infancy^69–71^. It is probably due to the fact that the infant is exposed to viruses or microorganisms expressing GalT in the gastrointestinal tract^72^. Because of this phenomenon, GalT expressed on the cell surface in pigs is the main hurdle causing hyperacute rejection in pig-to-human xenotransplantation. Antigen-antibodies mediated rejection still remains the main cause for xenotransplantation failure following knock-out of GalT. Other non-gal antigens, such as Neu5Gc or Sda, have been knocked out to decrease anti-pig antibody binding in human^9,73,74^. However, the survival of Neu5Gc deficient pig kidney was shorter than GTKO pig kidney in pig-to-nonhuman primate xenotransplantation, and antibody binding to Neu5Gc deficient pig were also increased in old world monkey because unlike humans, old world monkey have activated CMAH gene, and possess naturally-existing antibodies directed to Neu5Gc deficient pig^74–76^. Therefore, the current model of pig-to-non-human primate xenotransplantation is less than ideal given the expectation for improved survival in the first pig-to-human trials with a Neu5Gc deficient pig compared to the pig-to-non-human primate model. These findings and our results imply that triple knock-out of GGTA1, CMAH and β4GalNT2 and insertion of hCD55 and hCD39 genes could be effective to prevent immune rejection in pig-to-human xenotransplantation.

hCD55 plays a role in the regulation of complement activation, which is caused by the antigen–antibody complex via the classical pathway. The mechanism between hCD55 and NK cells is unknown, however hCD55 was associated with NK cell-mediated lysis, whereas other complement regulatory proteins including hCD46 and hCD59 were not^22^. In this study, we successfully eliminated the GalT epitope through targeting of the hCD55/hCD39 knock-in vector into the GGTA1 gene locus. Ablation of the GalT epitope considerably decreased human complement-mediated cytotoxicity and human antibody binding compared to wild-type pig cells. Although human antibody binding to cells from GTKO/CD55/CD39 pig was not greater than to cells from GTKO pigs, our transgenic pig cells showed further prevention against human complement-mediated lysis compared to GTKO pig cells (Fig. 2).

Decrease of C3 deposition on GTKO/CD55/CD39 pig cells following activation of the complement cascade was comparable to that in cells from GTKO, that also proved the inhibitory effect of the complement cascade by targeting hCD55 (Fig. 3A).

These data imply that the hCD55 gene targeted in our GTKO/CD55/CD39 pig could be capable of dramatically controlling the complement reaction in pig-to-human humoral rejection. Although precluding hyperacute rejection or complement activation, coagulation disorders also remain a barrier for successful pig-to-primate xenotransplantation. At the sites of vascular injury, platelet adhesion is initiated and activated by agonists, including ADP and collagen. Therefore, platelets, which have a role in the interaction between endothelium and leukocytes in the form of platelet microthrombi, have been thought to be a major player in the process of improving survival of organ xenotransplantation^77,78^. To regulate platelet-mediated thrombosis, we also aimed to knock-in hCD39 with hCD55. As a result, the platelet aggregation was much more delayed in PRP from GTKO/CD55/CD39 pig than PRP from wild-type and GTKO pigs, for not only ADP-induced, but also collagen-induced platelet aggregation (Fig. 2B). These results showed that our GTKO/CD55/CD39 pig could prevent not only humoral rejection but also platelet-mediated thrombosis in pig-to-primate xenotransplantation.

Our endogenous-mediated expression of transgenes by knock-in, targeting of hCD55 and hCD39 into the GGTA1 locus, induced abundant and stable expression of functioning proteins as well. Ablation of the GalT epitope and concomitant expression of hCD55 improved the inhibition of complement-mediated cytotoxicity compared to wild-type and GTKO pigs. The function of hCD39 was also proven by platelet aggregation analysis. Although hCD55 and hCD39 are not novel genes for xenotransplantation, endogenous promoter-mediated expression of hCD55 and hCD39 in a transgenic pig had not been tried in pig-to-non-human primate xenotransplantation. Moreover, our transgenic pig showed similar expression levels of hCD55 and higher expression of hCD39 compared to human aorta endothelial cells (Fig. 1D). In this phenomenon, our transgenic pig could be a comparable model to various transgenic pigs for xenotransplantation.

## Methods

### Ethical statements and animal carep

All experimental methods were carried out according to ARRIVE guidelines. All methods were performed in accordance with relevant guidelines and regulations. All protocols of animal care and use procedure were approved by the Institutional Animal Care and Use Committee of Optipharm, Inc., Life Science Institute (IACUC approval No. OPT-140103-1, D-grade). All pigs used in the experiments were white Yucatan miniature pigs and approved by institutional animal care. The animal facility for all pigs at Optipharm, Inc., was a specific pathogen-free environment. Filtered water and air, and sterilized feed were supplied, and the rooms were maintained at 24 °C ± 2 °C and 12 h light/12 h dark cycles. For experiments of this study, one-year-old healthy pigs, wild-type, GTKO, and GTKO/CD55/CD39 were humanely euthanized by intravenous injection of 2 mM/kg potassium chloride solution under general anesthesia. After a veterinarian certified the death of the pigs, their organs were harvested.

### Construction of hCD55/hCD39 knock-in vector and CRISPR/Cas9 vector for GalT

Two homologous arms (left and right arms) were designed to target exon4 of the GGTA1 gene as described in Fig. 1. These arms were amplified from pig genomic DNA by PCR. hCD55 and hCD39 fragments were synthesized from human cDNA by PCR. Left and right arm DNA fragments were inserted into modified PGKneolox2DTA.2 (Addgene plasmid 13449). hCD55 and hCD39 fragments were inserted between the left arm and the neomycin fragment. IRES sequences were inserted between the hCD55 and hCD39 fragments to express hCD39. Small guide RNA sequences (5’-AATGAATGTCAAAGGAAGAG-3’) for CRISPR/Cas9 vector were targeted for the ATG codon of the GGTA1 gene.

### Culture, transfection, and selection of pig primary ear skin fibroblasts

Ear skin fibroblasts were isolated from 2 month-old white yucatan miniature pigs. Isolated ear skin fibroblasts were cultured in Dulbecco's modified eagles medium (DMEM, Welgene, Korea) containing 15% fetal bovine serum (FBS, Hyclone, USA), 1% non-essential amino acid, 0.1 mM β-mercaptoethanol, and 1% antibiotic–antimycotic (Gibco, USA). Transfection occurred in 1 × 10^6^ suspended ear skin fibroblasts at 90% confluency, with 5 μg of GTKO/hCD55/hCD39 knock-in vector and 5 μg of CRISPR/Cas9 for GGTA1 using electroporation (Amaxa 4D, Lonza, USA), according to manufacturer’s instructions. After 2 days of transfection, the cells were incubated with neomycin (300 μg/mL) for further 14 days. After 14 days of transfection, the fibroblasts were harvested and stained with anti-hCD39 antibody (Santacruz, USA). CD39 positive cells were sorted by flowcytometry (Aria II, BD, USA).

### Production of cloned pig from SCNT and embryo transfer

SCNT and embryo transfers were performed as described previously in our study^37^. The pregnancy status was monitored with an ultrasound scanner (Mysono 201, Medison Co., LTD, Korea).

### Verification of targeted donor cells and transgenic pigs

From the single cell culture, 24 colonies were picked and 20 colonies were expanded to be analyzed. Each colony was harvested in 12-well plate culture vessels. Genomic DNA was extracted using DNeasy blood and tissue kit (QIAGEN, USA) from each colony and cloned pig tissue. Left arm, right arm, and long PCR amplification were performed to screen targeted colony and cloned pig using primers as described in supplementary Table S1.

### Expression of GalT, hCD55, and hCD39

For flow cytometry analysis, harvested cells were suspended in 100 µL of Dulbecco’s phosphate buffered saline (DPBS, Gibco) and stained with 2 µL of anti-hCD55 (sc-57133, Santacruz) or anti-hCD39 (sc-65232, Santacruz) for 2 h at room temperature. Fluorescein isothiocyanate (FITC)-conjugated secondary antibodies were then incubated for 1 h on ice to analyze the expression of hCD55 and hCD39. To analyze GalT expression, 2 µL of Alexa Flour 488®-conjugated isolectin-ib4 (I21411, Invitrogen, USA) was incubated with the suspended cells for 3 h on ice. Expression of GalT, hCD55, and hCD39 on the cell surface was estimated by flow cytometry (Calibur-S system, BD). To identify translational expression of hCD55 and hCD39, 2 × 10^6^ cells were harvested and lysed with 30 µL of mammalian protein extract reagent (Thermo, USA) for 4 h on ice. Proteins extracted from the cells were quantified using the Bradford assay (BioRad, USA) and resolved on 8% sodium dodecyl sulfate polyacrylamide gels. The proteins were then transferred to nitrocellulose membrane, which was blocked with 1X Tris-buffered saline, 0.1% Tween-20 containing 5% skimmed milk for 1 h at room temperature. Incubation with anti-hCD39 (sc-33558, Santacruz), or anti-hCD55 (ab54595, Abcam), or anti-beta-actin (sc-47778, Santacruz) as primary antibodies happened overnight at 4 °C. The membrane was then incubated with horseradish peroxidase-conjugated secondary antibodies for 1 h at room temperature. Human hCD55 and hCD39 were detected with the ECL chemiluminescence kit for western blot analysis (GE Healthcare, UK), according to the manufacturer’s protocol.

### Isolation of PBMCs and RBCs

A total of 5 mL of whole blood from wild-type, GTKO (developed in our previous study^79^), and GTKO/CD55/CD39 pigs was collected into EDTA tubes (BD). PBMC and RBCs were separated from collected blood using density media ficoll-paque plus (GE), according to manufacturer’s protocol. Isolated PBMCs and RBCs were washed with DPBS (Gibco), and immediately used for complement-mediated cytotoxicity or human antibody binding assays, or for confirmation of gene expression.

### Culture of Aorta endothelial cells, corneal endothelial cells, and kidney cells

Pig aortas were incubated with 0.005% collagenase type IV (sigma, USA), and the isolated cells were washed with DPBS(Gibco). The cells were then culture with endothelial cell growth medium-2 bullet kit (Lonza, Switzerland). Pig corneal endothelial cells were isolated and cultured, as described in our previous study^80^. Pig kidney tissues were minced into 1–2-mm sections, and digested with 3% collagenase type IV. The digested tissues were passed through a 100-m cell strainer (BD). The isolated cells were washed with DPBS (Gibco), and cultured in DMEM (Welgene) containing 15% FBS (Hyclone), 1% non-essential amino acid, 0.1 mM β-mercaptoethanol, and 1% antibiotic–antimycotic (Gibco).

### Complement-mediated cytotoxicity

2 × 10^5^ of PBMCs and RBCs isolated from pigs were incubated with 0%, 3.125%, 6.25%, 12.5%, and 25% of pooled human complement serum (Innovative research, USA) for 2 h (PBMCs) or overnight (RBCs) on v-bottom 96-well plates (Corning, USA). After incubation, human complement serum was removed following centrifugation. PBMCs were then re-suspended with 100 µL of RPMI 1640 media (Gibco) containing 5% fetal bovine serum (Gibco), 15 mM HEPES (Sigma), and 0.2 M EDTA (Bioneer, Korea). To estimate viability against human complement serum, 10 µL of CCK-8 solution was added to suspended PBMCs, and the absorbance of each well at 450 nm was measured. After incubation of RBCs with human complement serum, viability was estimated by measurement of the absorbance (405 nm) of the remaining viable RBCs.

### Human antibody binding assay

Pig PBMCs and RBCs were incubated with 10% normal human serum (Millipore, USA) for 30 min at 4 °C. PBMCs and RBCs were then washed by centrifugation and incubated with 100 μL of PBS containing 3 µL of anti-human IgG (62-8411, Invitrogen) or anti-human IgM (F5384, Sigma) for 1 h at 4 °C. Stained PBMCs and RBCs were analyzed using flow cytometry (CantoII, BD). Human antibody binding was calculated using the MFI formula below:

$$Relative \;MFI = \frac{{{\text{MFI }}\;{\text{of }}\;{\text{antibody}}\;{\text{ stained }}\;{\text{with }}\;{\text{human }}\;{\text{serum }}\;{\text{incubation}}}}{{{\text{MFI}}\;{\text{ of}}\;{\text{ antibody}}\;{\text{ stained }}\;{\text{cells }}\;{\text{without }}\;{\text{human }}\;{\text{serum}}\;{\text{ incubation}}}}$$

### C3 deposition

RBCs from wild-type, GTKO, and GTKO/CD55/CD39 pigs (5 × 10^5^) were incubated with human complement serum (Innovative technology, USA) at a concentration range of 0%, 25%, and 50% for 24 h. The cells were then incubated with an anti-C3 antibody (Santacruz) for 2 h at room temperature. The FITC-conjugated secondary antibody (Abcam) was applied for 1 h on ice. After each incubation, the cells were washed three times with 1 mL of PBS. Stained cells were analyzed by flow cytometry (CantoII, BD, USA).

### Platelet aggregation analysis

Blood was drawn from wild-type, GTKO and GTKO/CD55/CD39 pigs into trisodium citrate (0.109 M, 3.2%) tubes (BD). Pig platelet-rich plasma (PRP) and platelet-poor plasma (PPP) were separated by centrifugation. Platelet aggregation response against ADP (10 µM) and collagen (2 mg/mL) was measured by light transmission aggregometry (chrono-log, USA).

## Supplementary Information

Supplementary Legends.

Supplementary Information 2.

Supplementary Information 3.

Supplementary Information 4.

Supplementary Information 5.

Supplementary Information 6.

Supplementary Information 7.

## Data Availability

The datasets used and analysed during the current study available from the corresponding author on reasonable request.
