# Supplementary material for: A desirable transgenic strategy using GGTA1 endogenous promoter-mediated knock-in for xenotransplantation model
Source: Sci Rep. 2022 Jun 10;12:9611. doi: 10.1038/s41598-022-13536-z (PMC9187654; doi:10.1038/s41598-022-13536-z)
Supplement: Supplementary file 1 — Supplementary Legends. [file 41598_2022_13536_MOESM1_ESM.docx]

**Supplementary figure S1. Establishment of targeted donor cell. (A) Schematic of targeting vector to ablate GalT and knock-in of hCD55 and hCD39 gene. (B) CRISPR/Cas9 target sites for exon4 of GGTA1. (C) Selection of targeted cells (CD39 positive) by FACS sorting (Aria II, BD, USA). (D) Verification of targeted single clones by PCR analysis. (E) Expression of GT, CD55 and CD39 on targeted clone by FACS analysis (Calibur-S system, BD, USA, flowjo software V10.6.2, https://www.flowjo.com/solutions/flowjo/downloads) (Blue line : wild type; Red line : targeted colony). (F) Translational expression of targeted CD55 and CD39 gene by western blot analysis. The PCR products and protein expression of western blotting were captured using Biorad image analysis system (Chemidoc MP, Biorad, USA, Image Lab Touch software V3.0.1, https://www.bio-rad.com/ko-kr/product/image-lab-touch-software?ID=PJW3UUTU86LJ). The original agarose gels, and blots are presented in Supplementary figure S2.**

**Supplementary figure S2. the original agarose gels (A) blots (B) in supplementary fig. S1. (-) : wild-type, PC : positive control. anti-CD39 antibody (Santacruz; sc-33558; molecular weight : 70-100kDa; reactivity : human, mouse, rat), anti-CD55 antibody (Abcam; ab54595; molecular weight : 75-100kDa; reactivity : human), anti-Actin antibody (Santacruz; sc-1616; molecular weight : 40kDa). The PCR products and protein expression of western blotting were captured using Biorad image analysis system (Chemidoc MP, Biorad, USA, Image Lab Touch software V3.0.1, https://www.bio-rad.com/ko-kr/product/image-lab-touch-software?ID=PJW3UUTU86LJ).**

**Supplementary figure S3. the original agarose gels in fig. 1. lane 1 : targeted donor cell; lane 2 : wild-type; lane 3 : GTKO/CD55/CD39 pig. The PCR products were captured using Biorad image analysis system (Chemidoc MP, Biorad, USA, Image Lab Touch software V3.0.1, https://www.bio-rad.com/ko-kr/product/image-lab-touch-software?ID=PJW3UUTU86LJ).**

**Supplementary figure S4. Production of four live born GTKO/CD55/CD39 piglets. (A) Photographs and birth weight of GTKO/CD55/CD39 piglets. (B) targeting PCR of four live born piglets to verify GTKO/CD55/CD39 (lane 1 : Targeted donor; lane 2 : wild type; lane 3 : piglet 1; lane 4 : piglet 2; lane 5 : piglet 3; lane 6 : piglet 4). The PCR products were captured using Biorad image analysis system (Chemidoc MP, Biorad, USA, Image Lab Touch software V3.0.1, https://www.bio-rad.com/ko-kr/product/image-lab-touch-software?ID=PJW3UUTU86LJ). (C) Disruption of non-targeted GGTA1 allele by CRISPR/Cas9 in four live born piglets. The alignment of sequences was analyzed using BioEdit software V7.2 (https://bioedit.software.informer.com/7.2/). Three of live born piglets were weakly born and died within 3 weeks after birth. One piglet was survived and used for this study.**

**Supplementary figure S5. Off-site target analysis of gRNA for GGTA1 in GTKO/CD55/CD39 pig. Potential off-target sequences were identified by CRISPR RGEN Tools (http://www.rgenome.net/cas-offinder/) in pig genome, and were only 3 mismatches (3 MMs) and no 1 or 2 mismatches (MMs) off-target candidate sequences in pig genome. Box in black represented sequence of mismatches with on-target gRNA sequence. Box in red represented PAM sequence of each target site. The alignment of sequences was analyzed using BioEdit software V7.2 (https://bioedit.software.informer.com/7.2/).**

**Supplementary table S1. PCR primer list to verify targeting in this study.**
