# Supplementary figures and images for: A desirable transgenic strategy using GGTA1 endogenous promoter-mediated knock-in for xenotransplantation model
Source: Sci Rep. 2022 Jun 10;12:9611. doi: 10.1038/s41598-022-13536-z (PMC9187654; doi:10.1038/s41598-022-13536-z)

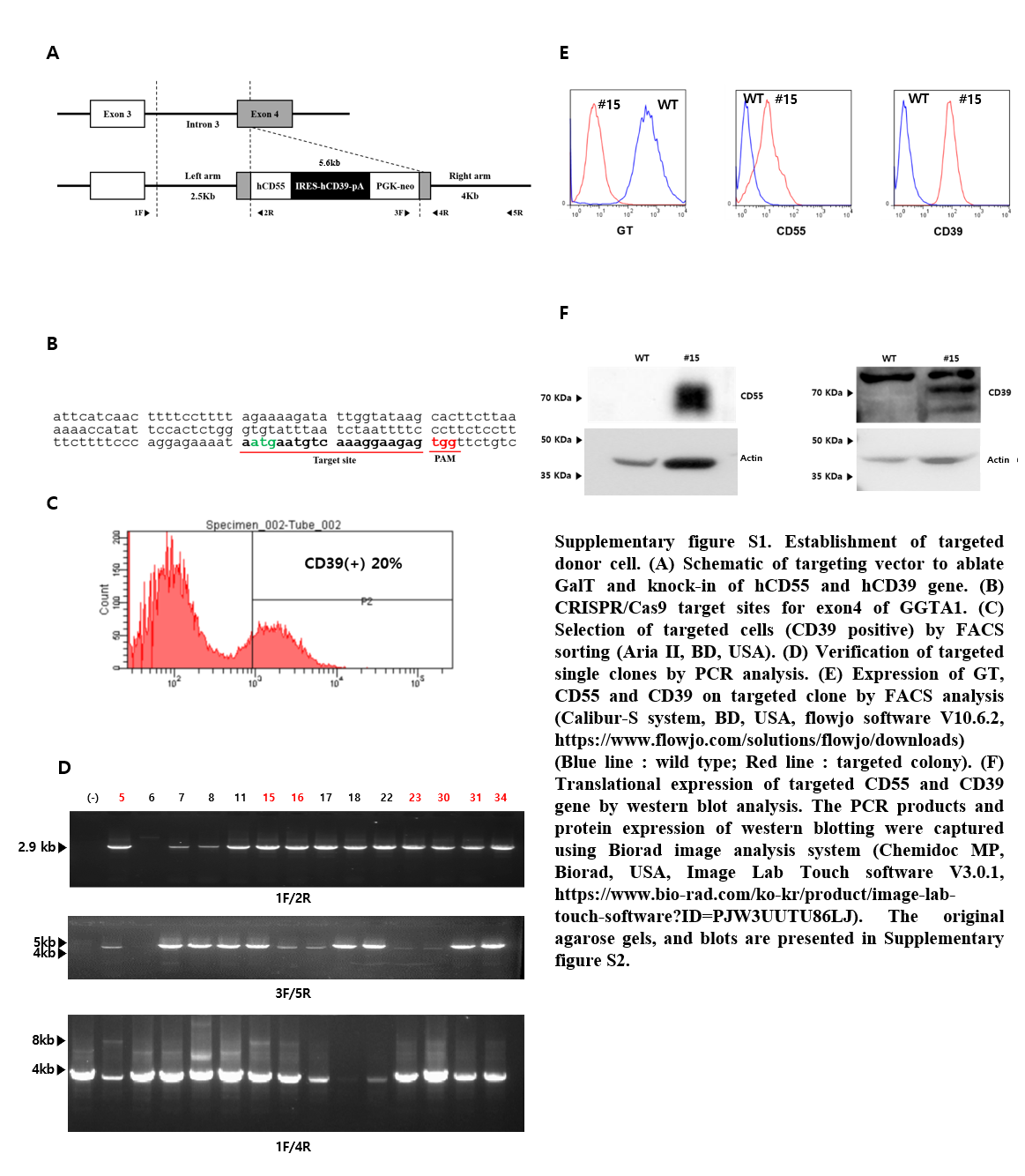

Supplement: Supplementary file 2 — Supplementary Information 2. [file 41598_2022_13536_MOESM2_ESM.tif]

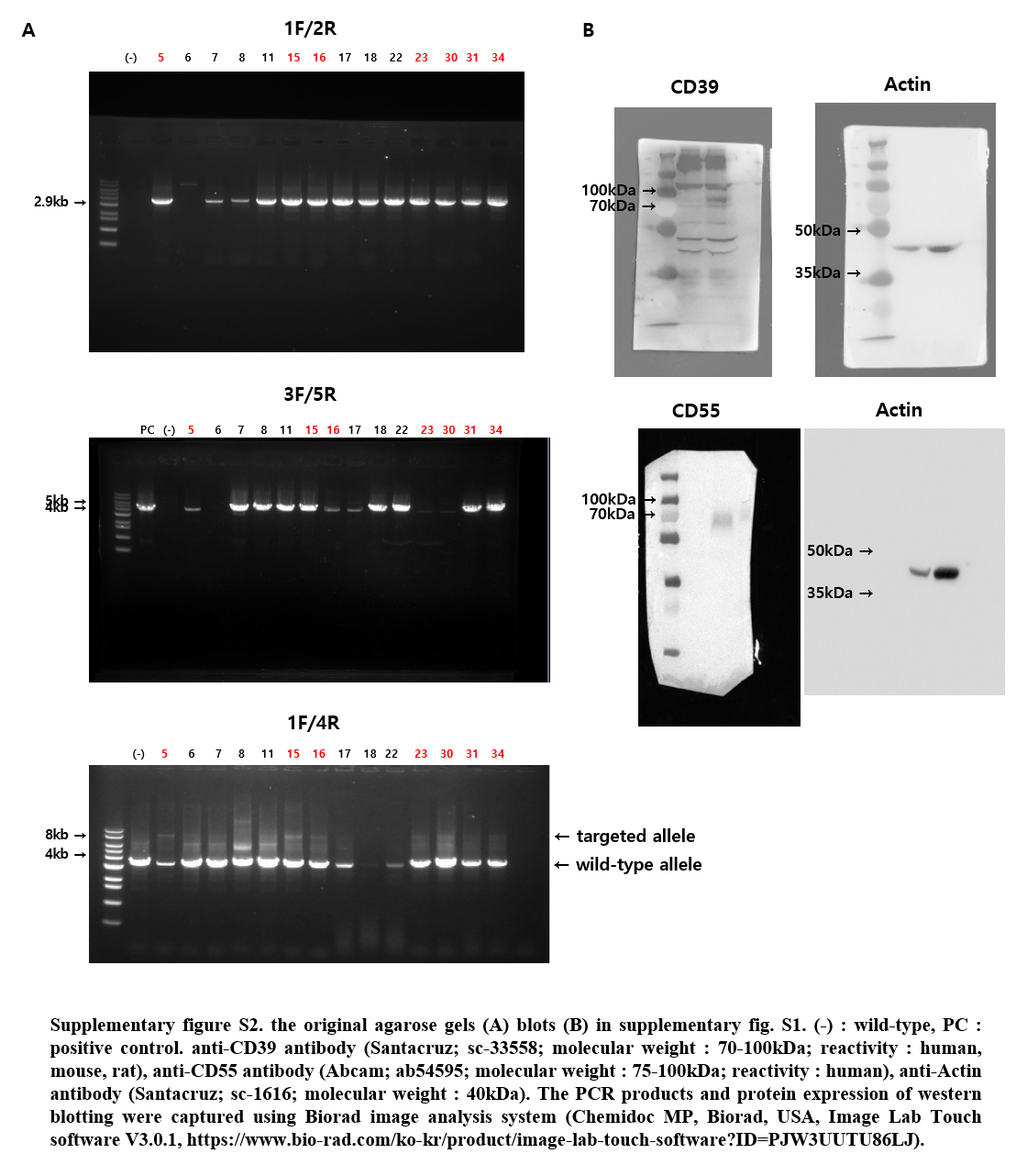

Supplement: Supplementary file 3 — Supplementary Information 3. [file 41598_2022_13536_MOESM3_ESM.tif]

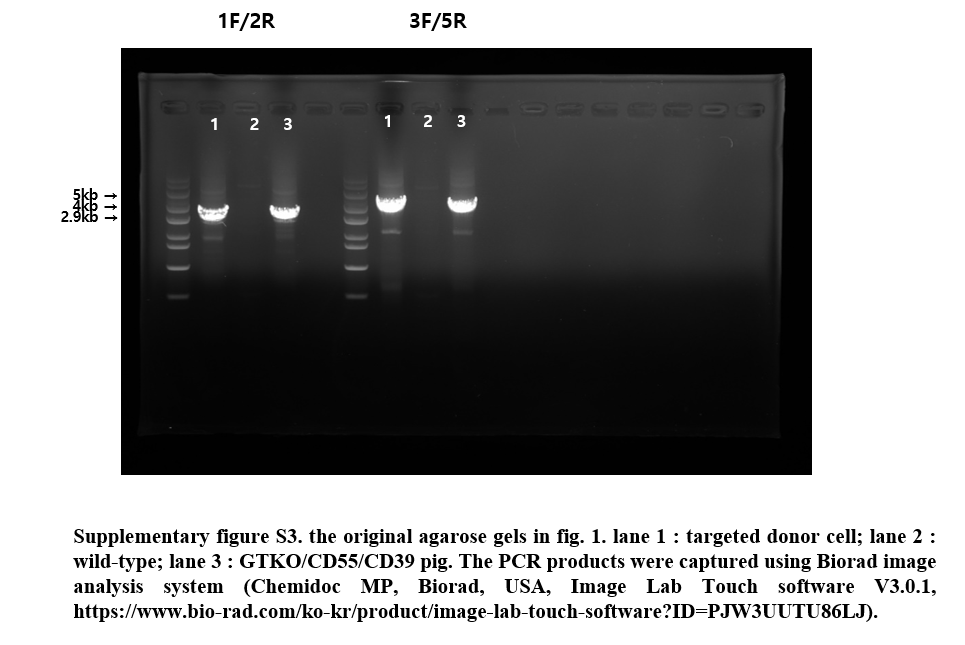

Supplement: Supplementary file 4 — Supplementary Information 4. [file 41598_2022_13536_MOESM4_ESM.tif]

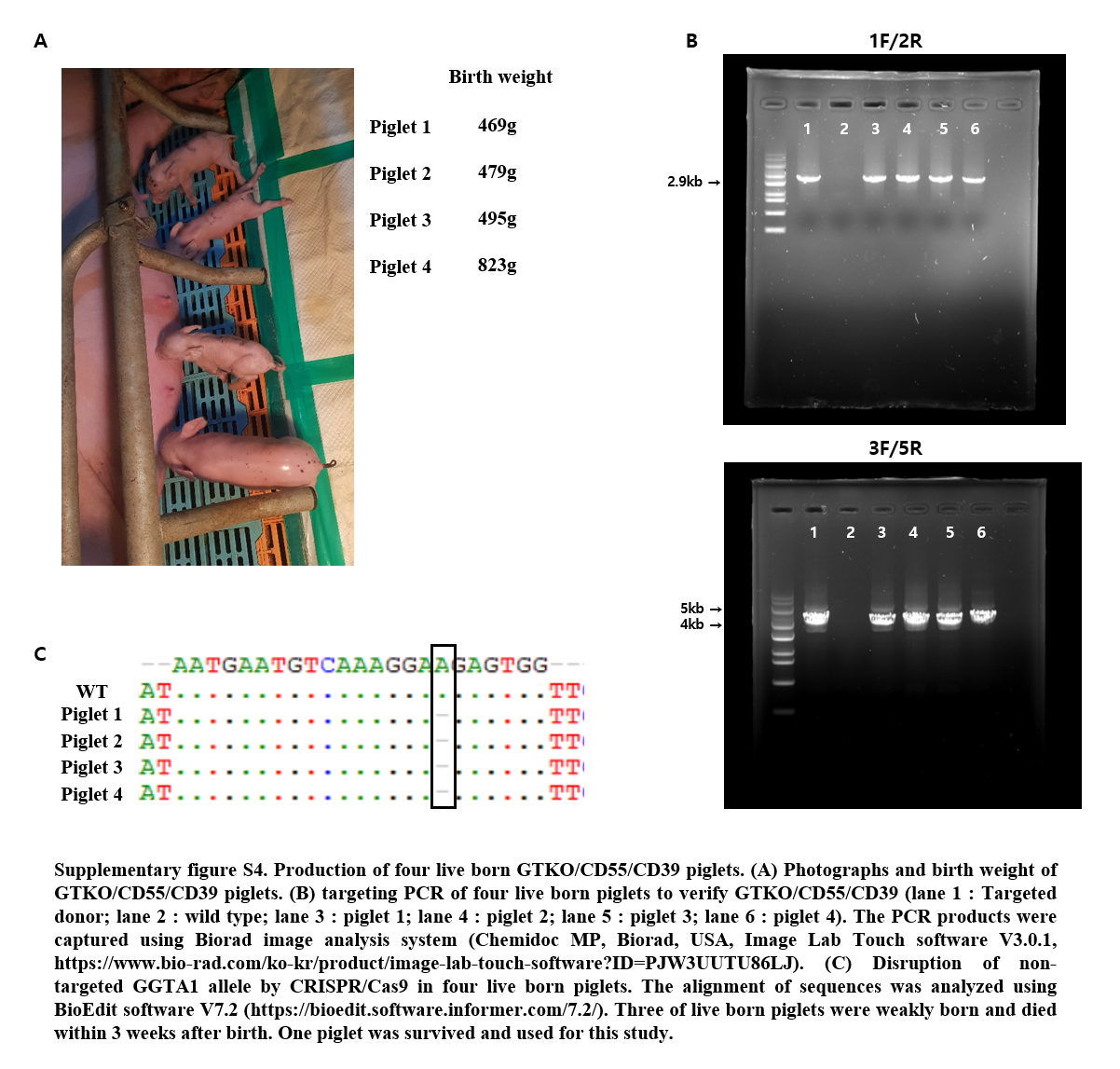

Supplement: Supplementary file 5 — Supplementary Information 5. [file 41598_2022_13536_MOESM5_ESM.tif]

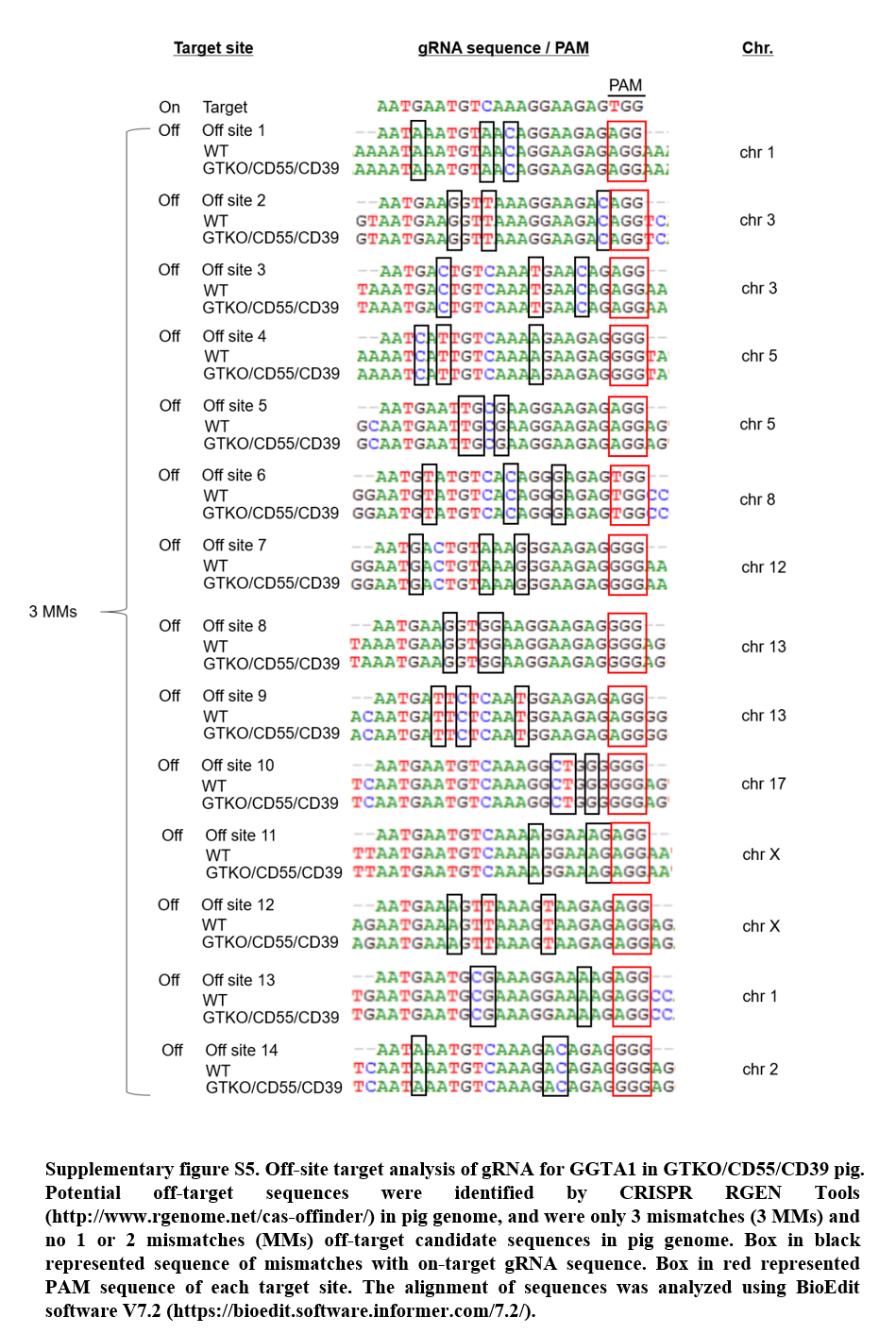

Supplement: Supplementary file 6 — Supplementary Information 6. [file 41598_2022_13536_MOESM6_ESM.tif]

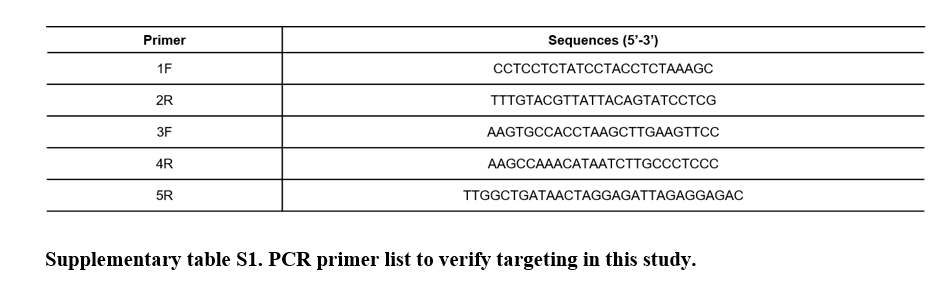

Supplement: Supplementary file 7 — Supplementary Information 7. [file 41598_2022_13536_MOESM7_ESM.tif]
